# Relationship between loneliness and academic procrastination with cyberspace addiction in single-parent adolescents’ academic

**DOI:** 10.3389/fpsyg.2026.1714353

**Published:** 2026-03-30

**Authors:** Yiping Zheng, Yanchao Feng

**Affiliations:** 1 School of Marxism, Shanghai Jiao Tong University, Shanghai, China; 2 Tianjin University of Commerce, Tianjin, China

**Keywords:** academic procrastination, cyberspace addiction, loneliness, single-parent adolescents, statistical approaches

## Abstract

**Background:**

Adolescents from single-parent families experience unique psychosocial challenges that increase their vulnerability to loneliness, academic procrastination, and problematic cyberspace use. Although these risks are well-documented individually, the interrelationships among these factors within this specific population remain inadequately understood.

**Objectives:**

This study examined the relationships between loneliness, academic procrastination, and cyberspace addiction among single-parent adolescents and assessed the extent to which these factors predict problematic cyberspace use.

**Methods:**

A correlational design was employed with 140 single-parent adolescents aged 15–18 years from Shanghai, recruited through purposive sampling. Participants completed the UCLA Loneliness Scale, the Academic Procrastination Scale, and Young’s Internet Cyberspace Test (1998). Data were analyzed using Pearson correlation and stepwise regression analysis.

**Results:**

The results revealed significant positive correlations between loneliness and cyberspace addiction (*r* = 0.760, *p* < 0.01) and between academic procrastination and cyberspace addiction (*r* = 0.561, *p* < 0.01). Stepwise regression analysis indicated that loneliness alone accounted for 33.1% of the variance in cyberspace addiction (R^2^ = 0.331). The inclusion of academic procrastination significantly enhanced the predictive model, with both factors together explaining 59.1% of the total variance (R^2^ = 0.591, ΔR^2^ = 0.260).

**Conclusion:**

Loneliness and academic procrastination serve as significant predictors of cyberspace addiction among single-parent adolescents. These findings underscore the importance of integrated intervention strategies in educational and clinical settings that simultaneously address emotional wellbeing and academic coping skills to mitigate problematic cyberspace use in this vulnerable population.

## Introduction

### Litrature review

The proliferation of single-parent families in modern societies significantly impacts adolescent development, creating unique vulnerabilities in social, emotional, and educational domains (Yorks, 2022). Approximately 90% of these families are mother-headed, facing compounded challenges of economic strain, limited social support, and emotional isolation that disproportionately affect adolescents (Tandywijaya et al., 2023). Research consistently demonstrates that adolescents in single-parent families experience elevated rates of behavioral and psychological difficulties, including aggression, academic underachievement, poor self-concept, and pronounced loneliness (Liu et al., 2024).

The evolution of research on single-parent family outcomes has progressed from descriptive accounts toward mechanistic explanations, revealing three convergent research clusters that inform the present investigation.

The first cluster examines broad psychosocial outcomes in single-parent families. Cross-cultural findings converge on the centrality of loneliness as a core feature of these adolescents’ lived experience (Liu et al., 2024; Yorks, 2022). However, this research tradition has largely identified general correlations without elucidating specific psychological mechanisms linking loneliness to maladaptive behaviors.

The second cluster investigates relationships between negative emotional states and problematic technology use. Two competing hypotheses have emerged: the “social compensation” hypothesis suggests cyberspace offers lonely adolescents opportunities to fulfill unmet relational needs (Jean Yeung and Park, 2016; Wang, 2024), whereas the “isolation exacerbation” hypothesis contends that superficial online interactions deepen loneliness, creating self-perpetuating cycles (Kent-Marvick et al., 2022; Qi et al., 2025).

Meta-analytic evidence indicates a moderate positive association between loneliness and internet/smartphone addiction among adolescents (pooled *r* = 0.252), supporting the relevance of this relationship while highlighting the need for further mechanistic clarification (Ge et al., 2023). Similarly, research among high school students reveals complex patterns wherein quantitative and qualitative findings may diverge, suggesting that the loneliness-technology relationship requires contextual interpretation (Can, 2023). Recent work further identifies hopelessness as a mediating mechanism in the social media addiction-loneliness relationship, pointing to the importance of examining indirect pathways (Durmuş et al., 2025).

The third cluster encompasses educational research, wherein academic procrastination has been reconceptualized from time-management deficit (Solomon and Rothblum, 1984) to a maladaptive emotion regulation strategy (Sirois and Pychyl, 2013). This theoretical advancement provides critical integration: individuals experiencing negative emotions may seek immediate gratification through alternative activities, including cyberspace engagement, to escape unpleasant states, resulting in delayed academic tasks (Kara and Gülbahçe, 2026; Nadarajan and Hengudomsub, 2023).

Despite theoretical progress within each cluster, a structural gap persists. Existing studies predominantly examine dyadic relationships within general adolescent samples, with limited integration of these constructs within coherent explanatory models situated in the specific at-risk context of single-parent families. The question remains: among single-parent adolescents, does loneliness increase cyberspace addiction risk through the mechanism of academic procrastination as a maladaptive emotion regulation strategy?

### Theoretical framework

This study integrates Attachment Theory (Bowlby, 1969) with the Cognitive-Behavioral Model of pathological internet use (Davis, 2001; Zalaznik et al., 2025). From an attachment perspective, adolescents in single-parent families may experience disruptions in secure base formation, fostering internal working models characterized by expectations of social unreliability (Saini and Hasan, 2024). This manifests as chronic loneliness—persistent perceived social isolation (Margareta, 2026). The cognitive-behavioral model posits that maladaptive cognitions and deficient self-regulation contribute to problematic internet use (Gao et al., 2022; Ravichandran and Anbu, 2026).

Cyberspace addiction—characterized by impaired control over usage and emergence of uncontrollable daily behaviors (Aktaş Terzioğlu and Büber, 2025). represents a particular concern for single-parent adolescents (Liu, 2023; Zhou et al., 2025). These adolescents face heightened susceptibility due to limited emotional support, spending increased time online navigating challenges stemming from parental absence (Dan et al., 2026).

Academic procrastination constitutes a determining factor in this dynamic (Nadarajan and Hengudomsub, 2023). Contemporary research emphasizes procrastination as short-term emotion regulation (Sirois and Pychyl, 2013), wherein individuals avoid tasks eliciting negative affect and seek immediate mood repair through alternative activities such as cyberspace engagement (Kara and Gülbahçe, 2026). Adolescents addicted to cyberspace allocate considerable time to non-academic online activities, diminishing academic motivation and concentration. This addiction functions simultaneously as an escape mechanism and a consequence of procrastination, diverting adolescents from responsibilities while providing immediate satisfaction that impedes task completion (Nadarajan and Hengudomsub, 2023). Such disruption heightens anxiety regarding unfinished assignments, reinforcing cyberspace engagement in a vicious cycle culminating in academic failure, diminished self-confidence, and exacerbated loneliness.

Research confirms significantly elevated loneliness among single-parent adolescents (Kent-Marvick et al., 2022), driving them toward online interactions in search of connection (Qi et al., 2025). However, transient online relationships fail to fulfill core emotional needs, potentially intensifying isolation and reinforcing addictive patterns (Kent-Marvick et al., 2022). The mediating role of hopelessness in similar relationships (Durmuş et al., 2025) underscores the importance of examining psychological mechanisms linking emotional states to behavioral outcomes.

Academic procrastination may serve as the crucial linking mechanism within this dynamic. Lonely adolescents procrastinate on academic tasks to avoid negative affect, subsequently turning to cyberspace for distraction. This triadic relationship connects loneliness to both procrastination and addiction vulnerability, with procrastinatory behaviors providing opportunities for compensatory online engagement (Gabriel et al., 2025). Thus, procrastination represents a behavioral pathway through which loneliness translates into elevated risk of problematic cyberspace use (Pourabbas et al., 2026).

Although prior research has separately examined relationships among these variables, a notable paucity of integrated studies simultaneously investigates these three constructs within the high-risk population of single-parent adolescents. Existing literature predominantly focuses on general adolescent populations or analyzes dyadic relationships. Furthermore, most studies lack explicit guidance from an integrative theoretical model connecting familial context (single-parenthood) to intrapersonal emotion (loneliness), maladaptive behavior (procrastination), and compensatory outcomes (cyberspace addiction). The potential role of academic procrastination in the loneliness-cyberspace addiction relationship within single-parent families—where unique intersections of emotional vulnerability and academic pressure converge—remains inadequately examined.

This study was guided by the following questions:

(1). Is there a significant relationship between loneliness and cyberspace addiction among single-parent adolescents?
(2). Is there a significant relationship between academic procrastination and cyberspace addiction among single-parent adolescents?
(3). To what degree do loneliness and academic procrastination, in combination, predict cyberspace addiction in single-parent adolescents?

## Materials and methods

The present study was an applied research study using a descriptive correlational design. This choice aligns with the primary research objectives, which are to examine the nature and strength of relationships between the variables of loneliness, academic procrastination, and cyberspace addiction, and to assess their predictive utility, without inferring causality. Also, all methods were performed in accordance with the relevant guidelines and regulations. The statistical population of the present study included all single-parent adolescents aged 15–18 in the city of Shanghai.

Given the specific and relatively limited nature of the target population, a purposive sampling method was deemed appropriate to efficiently identify and access eligible participants within school settings. The sample size was determined using the guideline by Tabachnick and Fidell (18) (*n* > 50 + 8 m), resulting in the selection of 140 single-parent adolescents. In order to conduct the present study, after obtaining the necessary permits from the institutes in the regions of Shanghai, eight secondary schools for girls and boys were selected, and by visiting these schools, 140 students (72 boys and 68 girls) were selected as single-parents.

Then, the Russell Loneliness Scale (UCLA) (Russell, 1996), the Academic Procrastination Questionnaire (Solomon and Rothblum, 1984), and the Cyberspace Addiction Test (IAT) (Young, 1998) were measured and evaluated for each participant. This study involved human adolescent participants and was conducted in accordance with the ethical standards outlined in the 1964 Helsinki Declaration and its later amendments. Prior to data collection, we consulted with the relevant institutional authorities regarding ethical requirements for non-interventional educational research. Given the study’s non-experimental, correlational design and its administration within regular school settings during standard class hours, formal ethics committee approval was not mandated by the participating institutions or the local educational authorities under whose jurisdiction the research was conducted.

However, recognizing the importance of protecting adolescent participants, we implemented comprehensive ethical safeguards throughout the research process:

(1). Informed consent: Written informed consent was obtained from the legal guardians of all adolescent participants after providing detailed information about the study’s purpose, procedures, potential benefits, and voluntary nature.
(2). Participant assent: Written assent was obtained directly from all adolescent participants themselves, ensuring their willingness to participate was independently documented.
(3). Right to withdraw: All participants and their guardians were explicitly informed of their right to withdraw from the study at any time without any negative consequences.
(4). Confidentiality and anonymity: The researcher ensured complete confidentiality of all collected data. Identifying information was anonymized through unique participant codes, and data were stored securely with access restricted to the research team.
(5). Data protection: All procedures for data handling complied with standard data protection principles to prevent unauthorized access.
(6). Institutional permission: Official coordination and permission were obtained from the Shanghai Regional Education Center to facilitate the participation and cooperation of schools within the region prior to accessing schools and recruiting participants.

The combination of guardian consent, participant assent, institutional authorization, and rigorous confidentiality procedures ensured that the rights and wellbeing of all adolescent participants were protected throughout the research process, consistent with ethical principles for research involving human subjects even in the absence of formal committee review.

Inclusion criteria included: Age (age range 15–18 years), single-parent adolescents (with one parent) were allowed to participate, and written and informed consent was obtained from the adolescents and legal guardians. Exclusion criteria also included distortion and failure to complete the questionnaires, psychological and physical problems, and withdrawal from the study.

## Research tools

### Russell Loneliness Scale (UCLA)

This scale was developed by Russell (1996) with 20 items to assess dissatisfaction with social relationships in two domains, including limited intimate relationships and an insufficient social network. Responses and scoring in this scale are based on a four-point Likert scale from 1 (never) to 4 (often), and the range of scores obtained is between 20 and 80. The average score is 50, and higher scores indicate greater severity of loneliness. Responses to items 1, 5, 6, 9, 10, 15, 16, 19, and 20 were reverse-scored. Russell (1996) reported the internal consistency coefficient of this tool as 0.94. The reliability coefficient of this questionnaire was also estimated as 0.74 in the study by Dehkordi et al. (2015) using Cronbach’s alpha. Bhuiya et al. (2025) estimated the reliability of the overall instrument using Cronbach’s alpha at 0.69, with subscale values ranging from 0.63 to 0.74. The scale’s validity for use with Chinese students has been supported in prior research (Fan et al., 2024). In the present study, reliability was calculated via Cronbach’s alpha, yielding a value of 0.721 for the overall score and values ranging from 0.879 to 0.990 for the subscales.

### Academic procrastination questionnaire

This questionnaire was designed by Solomon and Rothblum (1984) in 1984 consists of 27 items that examine three components: preparing for exams (eight questions), preparing for assignments (11 questions), and preparing for term papers (eight questions). Participants show their level of agreement with each question by selecting one of five response options: “never,” “rarely,” “occasionally,” “most of the time,” and “always” which are assigned scores from 1 to 5, respectively. Also, some items (2, 4, 6, 11, 13, 15, 16, 21, 23, and 25) are reverse-scored. The reliability of this questionnaire has been verified in various studies, including (Durán-González et al., 2023), who obtained a reliability coefficient of 0.87 using Cronbach’s alpha method and confirmed its content validity. Cao et al. (2025) also estimated the internal reliability of the instrument using Cronbach’s alpha coefficient at 0.79. Its use in Chinese student studies has been documented (Tian et al., 2021), indicating its relevance to this cultural setting. In this work, Cronbach’s alpha was determined as 0.90 for the overall questionnaire score and ranged from 0.72 to 0.88 for the subscales.

### Young cyberspace addiction test

This questionnaire was developed by Young (1998) in 1998 and is one of the most valid questionnaires related to cyberspace addiction, which aims to measure the level of cyberspace addiction in different individuals. The questions of this test were designed based on the DSM for pathological gambling disorder, because it is believed that cyberspace addiction has a lot of similarities with gambling behavior. This scale examines behavioral problems, the degree of employment, compulsive use, emotional fluctuations, and the effects of cyberspace dependence. It has 20 questions, and its response range is five-choice Likert (1: rarely to 5: always). The range of scores is from 20 to 100; the higher this score, the more addicted the person is to cyberspace and vice versa. This standardized questionnaire and previous studies have confirmed its validity and reliability, yielding a Cronbach’s alpha of 0.90, and Lu et al. (2022) have confirmed its reliability with a Cronbach’s alpha of 81.00. Martín Herrero et al. (2024) reported reliability coefficients of 0.74 using Cronbach’s alpha and 0.75 using the split-half method for this test. In this work, the scale’s reliability, determined via Cronbach’s alpha coefficient, was 0.76 for the overall scale.

## Results

Demographic information of the research samples is reported and indicates that the overall sample of the study included 140 single-parent adolescents aged 15–18 years in Shanghai, with a mean age of 16.30 and a standard deviation of 1.192. 82 (58.6%) of the sample were in the age range of 15–16 years, and 58 (41.4%) of the sample were in the age range of 17–18 years. The gender of the subject group included 67 boys (47.9%) and 73 girls (52.1%).

The educational level of the sample group was 45 (32.1%) in the 9th grade, 26 (18.6%) in the 10th grade, 35 (25%) in the eleventh grade, and 34 (24.3%) in the 12th grade.

Regarding the guardianship status of the sample group, it was reported that 43 people (30.7%) were fathers, 63 people (45%) were mothers, and 34 people (24.3%) were other guardians. A total of 61 people (43.6%) had two family members, 36 people (25.7%) had three family members, and finally 43 people (30.7%) had four family members. A total of 15 people (10.7%) of the sample group reported a history of a disorder, and 125 people (89.3%) did not report a history of having a psychological disorder. Regarding the socio-economic status, the number of sample people (105 people; 75%) was in an average situation. Also, two people (14.3%) were in a poor situation, eight people (5.7%) were in a good situation, and seven people (5%) were in a very good situation. And finally, the component of parental education showed that 12 people (8.6%) were illiterate, 96 people (68.6%) had a diploma or less, 25 people (17.9%) had an associate’s degree or bachelor’s degree, and finally seven people (5%) had a master’s degree or higher.

Below, in Table 1, a summary of the descriptive statistics indicators of the subjects’ scores in each of the variables is presented.

**TABLE 1:** Descriptive statistics such as means, standard deviations, and etc., of the research variables.

Variables/statistical index	Minimum	Maximum	Mean	Standard deviation	Skewness	Kurtosis
Cyberspace addiction	20	85	46.11	16.97	0.423	−0.965
General loneliness	20	79	47.21	14.15	0.215	−1.141
Lack of social network	9	38	22.80	8.60	0.2561.451	−1.451
Lack of intimate relationships	10	42	23.72	8.20	0.162	−1.255
Academic neglect	33	97	60.96	22.08	0.352	−1.680
Preparing for exam	10	38	23.87	7.37	−0.226	−1.312
Preparing for homework	16	40	26.69	5.62	0.084	0.903
Preparation for end-of-semester papers	12	35	23.40	6.06	−0.319	−1.296

As is clear from Table 1, the descriptive indices of each of the research study variables are presented separately in the dimensions of the minimum and maximum range of scores, as well as the mean and standard deviation of each component and subscale. Table 1 also shows that the obtained skewness amount for the research study variables is in the range (2, −2). This means that the research study variables are normally related to skewness, and a symmetric distribution was observed. Their kurtosis amount is also in the range (2, −2). This indicates that the variable distribution is normal in terms of skewness and kurtosis. Pearson correlation analysis was employed to examine the relationship between loneliness and cyberspace addiction, with the results presented in Table 2. Significant positive correlations were observed between cyberspace addiction and both components of loneliness: lack of social network (*r* = 0.793, *p* < 0.01) and lack of intimate relationships (*r* = 0.799, *p* < 0.01). Furthermore, the overall loneliness score demonstrated a significant positive correlation with cyberspace addiction (*r* = 0.760, *p* < 0.01). These findings indicate that higher levels of loneliness, particularly deficits in social networks and intimate relationships, are associated with increased cyberspace addiction among single-parent adolescents. Notably, the strongest correlation emerged for lack of intimate relationships (*r* = 0.799).

**TABLE 2:** Correlation matrix between loneliness and cyberspace addiction.

Variable	1	2	3	4
Cyberspace addiction	–	–	–	–
Lack of social network	0.793**	–	–	–
Lack of intimate relationships	0.799**	0.782**	–	–
General loneliness	0.760**	0.771**	0.794**	–

**P < 0.01.

Pearson correlation analysis was conducted to examine the relationship between academic procrastination and cyberspace addiction, with the results presented in Table 3. As shown in Table 3, significant correlations were found between cyberspace addiction and academic procrastination, including its three components: preparing for exams, preparing for homework, and preparing for end-of-semester papers. Specifically, significant negative correlations were observed between cyberspace addiction and all three procrastination components: preparing for exams (*r* = −0.586, *p* < 0.01), preparing for homework (*r* = −0.560, *p* < 0.01), and preparing for end-of-semester papers (*r* = −0.585, *p* < 0.01). These negative relationships indicate that higher scores on these preparatory components—reflecting lower procrastination in these areas—are associated with lower levels of cyberspace addiction, and conversely, lower scores (indicating greater procrastination) are associated with higher cyberspace addiction. Additionally, a significant positive correlation emerged between overall academic procrastination and cyberspace addiction (*r* = 0.561, *p* < 0.01), indicating that adolescents with higher levels of academic procrastination tend to report greater cyberspace addiction.

**TABLE 3:** Correlation matrix of the variable of loneliness and cyberspace addiction.

Variable	1	2	3	4	5
Cyberspace addiction	–	–	–	–	–
Preparing for exam	−0.586**	–	–	–	–
Preparing for homework	−0.560**	0.522**	–	–	–
Preparation for end-of-semester papers	−0.585**	0.585**	0.504**	–	–
Academic procrastination	0.561**	−0.564**	−0.544**	−0.595**	–

**P < 0.01.

Next, the role of loneliness and procrastination in predicting cyberspace addiction in single-parent adolescents was examined. First, the parametric or non-parametric nature of the data was examined using the Shapiro–Wilk test to examine the normality of the data distribution, and the results are provided in Table 4.

**TABLE 4:** Kolmogorov-Smirnov test to check data normality, test of error independence, and linearity indices.

Variables	Kolmogorov-Smirnov test				Test of independence of errors	Testing the linearity indices of the model	
	*n*	*M*	Statistc (W)	*P*	Durbin-Watson	Tolerance	VIF
Cyberspace addiction	140	46.11	0.982	0.052	2.035	6.645	0.897
General loneliness	140	47.21	0.987	0.148	1.913	9.876	0.570
Academic procrastination	140	60.96	0.983	0.061	1.765	8.675	0.632

A non-significant Shapiro-Wilk test indicates no significant deviation from normality.

According to the results of the decision criterion (*P*-value), this value is greater than the significance level of 0.05. Therefore, there is no reason to reject the assumption that the sample in question is obtained from a normal distribution. In this sense, the research variables have a normal distribution.

Based on the results of Table 4, the tolerance coefficient and variance inflation index of the research variables are greater than 0.01 and smaller than 10, respectively. This indicates that the phenomenon of collinearity has not occurred in the research variables. In the following, the use of the Durbin-Watson index is presented to evaluate whether the assumption of independence of errors among the predictor variables is established or not. The value of each index is presented separately.

Therefore, in accordance with the view of Fylde, the assumption of independence of errors also holds among the research data. It is necessary to explain that the analysis of the data related to the Mehlno-Bais distance (D) showed that the data related to none of the participants formed multivariate outliers, and therefore it was concluded that the data distribution was normal. Next, to investigate the role of loneliness and academic procrastination in predicting coronavirus anxiety, multiple regression analysis was used using the stepwise method.

As presented in Table 5, the overall regression model was statistically significant, *F*(2, 137) = 265.34, *p* < 0.001. This indicates that the combination of loneliness and academic procrastination provides a significant explanation for the variance in cyberspace addiction scores. In the first step, loneliness alone entered the model, accounting for a substantial 33.1% (R^2^ = .331) of the variance in cyberspace addiction. In the second step, the addition of academic procrastination significantly improved the model, with the two predictors together explaining 59.1% (R^2^ = .591) of the total variance in cyberspace addiction. This change represents an increase of 26 percentage points in explanatory power (ΔR^2^ = .260).

**TABLE 5:** Stepwise regression analysis in predicting cyberspace addiction.

Model	SS	DF			MS		F	*P*
Regression	31847.257	2			15924.129		265.340	0.001
Residual	8221.914	137			60.014			
Total	40070.171	139			–			
**Predictor variables**	**Step**	***R***	**R^2^**	**ARS**	**Unstandardized coefficients**		**Beta**	**T (p)**
					**SE**	**B**		
Constant	–	–	–	–	2.293	−1.68	0.861	0.465 (0.873)
Loneliness	1	0.571	0.331	0.739	8.677	0.553	0.461	6.014 (0.001)
Loneliness × procrastination	2	0.769	0.591	0.792	7.747	0.356	0.463	8.17 (0.001)

An R^2^ of 0.591 indicates that approximately 59% of the differences (variance) in cyberspace addiction levels among the sampled single-parent adolescents can be predicted by—or attributed to—the combined influence of their levels of loneliness and academic procrastination. This is considered a large effect size in behavioral sciences, underscoring the strong, practical relevance of these two psychological factors in understanding the risk for cyberspace addiction within this specific population. The remaining 41% of the variance is associated with other factors not measured in this study.

## Discussion

The findings of the present study revealed a strong and significant positive relationship between loneliness and cyberspace addiction among single-parent adolescents. This result, consistent with previous research (Dong et al., 2024; Sarıalioğlu et al., 2022), can be interpreted within the study’s integrated theoretical framework. From the perspective of Attachment Theory, the single-parent family context may impair the formation of a secure base, fostering internal working models characterized by expectations of emotional and social instability (Saini and Hasan, 2024). These cognitive patterns predispose the individual to perceive the social world as unreliable, a state that manifests as chronic loneliness and persistent emotional distress (Weiss, 1973). Subsequently, based on the Cognitive-Behavioral Model (Davis, 2001; Gao et al., 2022), the adolescent, in an attempt to regulate this distressing negative affect, may develop maladaptive cognitions such as “I am only accepted online” or “No one understands me in the real world” and seek out cyberspace as an accessible and seemingly safe refuge to fulfill unmet relational needs and temporarily restore a sense of belonging. This supports the “social compensation” hypothesis (Jean Yeung and Park, 2016; Wang, 2024). However, the notably high strength of this relationship also warns of the risk of entrapment in an “isolation exacerbation” cycle, where superficial and transient online interactions ultimately fail to satisfy deep-seated needs for intimacy. By displacing real relationships and disrupting daily functioning, such use can intensify the initial loneliness and reinforce pathological dependence on cyberspace (Kent-Marvick et al., 2022; Qi et al., 2025).

Furthermore, the findings indicated a significant positive relationship between academic procrastination and cyberspace addiction. This aligns with the results of other studies (Balios et al., 2024; Gupta et al., 2024; Pérez-Jorge et al., 2024).

Interpreting this relationship based on the study’s theoretical framework moves beyond explanations centered solely on time management. In accordance with contemporary perspectives, academic procrastination is recognized as a maladaptive short-term emotion regulation strategy (Sirois and Pychyl, 2013). For the single-parent adolescent experiencing loneliness, challenging academic tasks can become anxiety-provoking stimuli that evoke feelings of inadequacy, hopelessness, and additional emotional burden. In this state, procrastination serves as a classic avoidant behavior to temporarily escape this negative affect and achieve immediate relief (Nadarajan and Hengudomsub, 2023). This avoidance, in turn, provides a powerful behavioral opportunity and cognitive justification for engaging in a more immediately rewarding and attractive alternative activity—namely, cyberspace—which offers a wide range of rapid reinforcements such as social validation, endless entertainment, and a false sense of connection (Gupta et al., 2024). Thus, cyberspace becomes not only the destination of this avoidance but also an efficient tool for fulfilling the same maladaptive emotion regulation function.

A crucial finding is the strong and shared explanatory power of these two variables. Regression analysis showed that while loneliness alone was a powerful predictor, adding academic procrastination to the model significantly increased its explanatory power, with the two variables together predicting 59.1% of the variance in cyberspace addiction. This finding empirically confirms the study’s proposed theoretical model, in which procrastination functions as a key mediating behavioral pathway. Accordingly, feelings of loneliness (with potential roots in attachment experiences) increase the tendency to use procrastination as an emotion regulation avoidance mechanism. This procrastinatory behavior, in turn, creates direct time, behavioral opportunity, and psychological justification for increased compensatory engagement with cyberspace, which can lead to problematic use patterns and ultimately addictive dependence (Gabriel et al., 2025; Qi et al., 2025). This mediating pathway clarifies the why and how behind the transformation of a distressing emotional state (loneliness) into a destructive behavioral outcome (cyberspace addiction) within the context of the dual emotional and academic pressures faced by single-parent adolescents.

Finally, these findings can be understood within a self-reinforcing vicious cycle. The initial pathway of loneliness → procrastination → compensatory cyberspace use can, through its consequences, intensify the initial conditions and create a positive feedback loop. Excessive and unregulated use of cyberspace may lead to neglect of academic duties, decreased performance and academic failure, as well as withdrawal from real-world social interactions. These outcomes, in turn, can trigger feelings of shame, inadequacy, greater social isolation, and consequently, a significant exacerbation of loneliness and a deeper decline in self-esteem (Alvia et al., 2024). This process establishes a persistent vicious cycle in which cyberspace addiction is both a consequence of the initial vulnerable conditions (loneliness and academic pressure) and a causal factor contributing to the perpetuation and deepening of those very conditions (Isa et al., 2024). Breaking this cycle requires interventions that simultaneously address both the emotional origin (loneliness) and the behavioral mechanism (procrastination).

Several methodological limitations warrant careful consideration. First, the cross-sectional design precludes causal inferences, as bidirectional relationships are equally plausible: while loneliness may drive cyberspace addiction, excessive online engagement could also exacerbate loneliness by displacing meaningful face-to-face interactions. Similarly, the relationship between academic procrastination and cyberspace addiction may be reciprocal, with each behavior reinforcing the other over time. Second, the reliance on self-report questionnaires introduces potential social desirability bias, particularly regarding sensitive topics like loneliness and problematic internet use. Furthermore, common method bias—variance attributable to measurement methods rather than the constructs themselves—may have artificially inflated the observed correlations. Although we employed well-validated instruments, the absence of temporal separation between measurements and lack of multi-informant data mean that method-induced inflation cannot be definitively ruled out.

Third, our analysis did not include statistical controls for potential confounding variables. While loneliness and academic procrastination demonstrated strong predictive power, unmeasured third variables—including personality traits (e.g., neuroticism, conscientiousness), family dynamics (e.g., parental monitoring, relationship quality with the custodial parent), socioeconomic resources, and pre-existing emotion regulation capacities—may confound the observed associations. The strong correlations reported (e.g., *r* = 0.86) could partially reflect shared variance with these unmeasured factors rather than unique relationships among the constructs of interest. An important alternative explanation concerns potential measurement overlap and construct confounding, as these instruments may tap shared underlying vulnerabilities such as negative affectivity, poor self-regulation capacity, or maladaptive coping styles rather than entirely distinct phenomena.

Fourth, the sample’s restriction to single-parent adolescents in Shanghai, China, significantly limits generalizability. Shanghai represents a unique urban context characterized by intense academic pressure, widespread digital infrastructure, and collectivist cultural values that may shape the expression of loneliness and technology use differently than in other settings.

Future longitudinal research with multiple time points, behavioral measures (e.g., actual screen time monitoring), and more rigorous statistical controls—including hierarchical regression analyses accounting for demographic and psychological covariates—is necessary to establish temporal precedence, reduce method bias, and isolate the unique contributions of loneliness and academic procrastination. Additionally, structural equation modeling with latent variables could help disentangle construct-specific variance from shared method variance and common underlying vulnerabilities. Cross-cultural comparative research with diverse samples is also needed to determine the extent to which these findings generalize across different populations and contexts.

## Conclusion

The present study confirms significant positive associations between loneliness, academic procrastination, and cyberspace addiction among single-parent adolescents. The primary contribution of this research lies in simultaneously examining these interrelated psychosocial risk factors within this specific vulnerable demographic, where emotional and academic pressures may uniquely converge. Our findings suggest that loneliness and procrastination are not merely individual correlates but may form a synergistic cluster that substantially predicts vulnerability to problematic internet use in this group.

These results underscore a critical need for integrated intervention strategies. In educational and clinical settings, practitioners should consider dual-pathway approaches that:

(1). Address emotional wellbeing: Implementing school-based programs to reduce social isolation and build peer support networks for adolescents from single-parent families.
(2). Enhance academic coping: Providing skills training in time management, task initiation, and study strategies to mitigate procrastination as a maladaptive coping mechanism.
(3). Promote digital literacy: Educate adolescents and their parents about healthy online habits, recognizing early signs of addictive use, and developing alternative offline activities for stress relief and connection.

For future research, longitudinal designs are essential to disentangle the directionality of these relationships and test causal pathways. Furthermore, investigating the role of potential moderators, such as the quality of the parent-adolescent relationship, specific family dynamics, or access to social support, would provide a more nuanced understanding of resilience and risk. This study serves as a foundation for developing targeted, evidence-based prevention and support frameworks tailored to the complex needs of single-parent adolescents.

## Data Availability

The original contributions presented in this study are included in this article/supplementary material, further inquiries can be directed to the corresponding author.
